# *Varroa destructor* relies on physical cues to feed in artificial conditions[Fn FN1]

**DOI:** 10.1051/parasite/2023049

**Published:** 2023-11-12

**Authors:** Vincent Piou, Caroline Vilarem, Solène Blanchard, Catherine Armengaud, Philipp Heeb, Angélique Vétillard

**Affiliations:** 1 Laboratoire Évolution et Diversité Biologique, UMR5174, CNRS-Université de Toulouse III-IRD, Université Paul Sabatier 31077 Toulouse France; 2 M2i Biocontrol–Entreprise SAS 46140 Parnac France; 3 Conservatoire National des Arts et Métiers (CNAM), Unité Métabiot 22440 Ploufragan France

**Keywords:** Artificial conditions, Olfaction, Physical cues, Shape, Pulse, Varroa destructor

## Abstract

Olfaction is a major sense in *Varroa destructor*. In natural conditions, it is known that this honey bee parasite relies on kairomones to detect its host or to reproduce. Yet, in artificial conditions, the parasite is able to feed and survive for a few days even though most honey bee pheromones are lacking. Other key cues are thus probably involved in *V. destructor* perception of its close environment. Here, we used several artificial feeding designs to explore the feeding behaviour of the parasite when it is deprived of olfactory cues. We found that *V. destructor* is still able to feed only guided by physical cues. The detection of the food source seems to be shape-related as a 3D membrane triggers arrestment and exploration more than a 2D membrane. The tactile sense of *V. destructor* could thus be essential to detect a feeding site, although further studies are needed to assess the importance of this sense combined with olfaction in natural conditions.

## Abstract (fr)

L’olfaction est un sens prépondérant chez *Varroa destructor*. En conditions naturelles, ce parasite de l’abeille domestique dépend en effet de kairomones qui lui permettent de détecter son hôte ou de se reproduire. Pourtant, lorsqu’il se retrouve en conditions artificielles, le parasite se nourrit et survit plusieurs jours malgré l’absence de la majorité des phéromones émises par l’abeille. Des indices clés autres qu’olfactifs sont donc très probablement impliqués dans la perception de l’environnement de *V. destructor*. Dans cette étude, plusieurs dispositifs d’alimentation artificielle ont été testés afin d’explorer le comportement de nourrissage du parasite lorsqu’il est privé d’indices olfactifs. Les résultats montrent que *V. destructor* est tout à fait capable de se nourrir en étant uniquement guidé par des indices physiques. En l’occurrence, la détection de la source nutritive semble être liée à sa forme puisqu’une membrane 3D provoque des comportements exploratoires plus prononcés qu’une membrane plane (2D). Le sens du toucher serait donc essentiel à *V. destructor* pour trouver son site de nourrissage. Des études complémentaires permettraient néanmoins d’évaluer les importances relatives des sens olfactif et tactile en conditions naturelles.

## Introduction

The foraging and feeding behaviour of mites remains poorly studied although it is of great interest in plant, animal and human health [23]. Through the predation of pests [7] or through the parasitism of plant and animal hosts [14, 62], mites can have significant agroecological impacts [23, 66]. Many cues are used by mites to find and feed on their host/prey, among which CO_2_, vibrations and pheromones have been shown to be relevant [13, 22, 39, 41, 56, 70]. Since many mites from the Parasitiformes super-order are blind, olfaction is a crucial sense for them to perceive the environment [24, 25, 39]. The effect of olfactory cues has been studied in mites from the genus Phytoseiidae such as in *Phytoseiulus persimilis*, which play an important role as biocontrol agents [2, 32–34, 69]. In these predatory mites, the detection of kairomones emitted by both their phytophagous prey and the wounded plant was shown to impact mite orientation [9, 58]. Although physical cues tend to be overlooked, several studies have highlighted the role they can play on mites [18, 22]. More specifically, heat, CO_2_ and vibrations are known to trigger host finding behaviours in several tick or mite species [1, 17, 41, 65]. In *Varroa destructor* (Parasitiformes, Mesostigmata), as in many other species, both chemical and physical cues can be detected by the parasite, allowing it to synchronise its lifecycle to the ecology of its hosts [10, 35, 45].

*Varroa destructor* is the main ectoparasite of the western honey bee *Apis mellifera*. Adult females feed on larval, pupal and adult bees, and transmit viruses in the process [60, 63]. Along with pesticide use, it is one of the main factors associated with colony losses in the Northern hemisphere [37]. Its life cycle is well described in the literature, although many behavioural aspects are not yet fully understood [11, 52]. As in Phytoseiid mites, olfaction seems to be the most important sense used by *V. destructor* [16, 28, 35, 51]. Many semiochemicals directly emitted by the bee, or coming from the colony matrices can be attractive to the parasite. For instance, specific blends of fatty acid esters from old bee larvae [6, 28] or other kairomones from cocoons and brood food [12, 36, 45] were shown to trigger arrestment (*i.e.*, an undirected change of locomotor activity as defined by Kennedy [27]) or the attraction of female *V. destructor*. The foreleg has been described as the major sensory organ in *V. destructor*, with the sensory pit concentrating chemo, thermo and hygroreceptors [10]. By blocking them mechanically, males are disoriented and females become unable to find a suitable host [10, 38].

Due to the significance of chemical factors in the parasite’s lifecycle, other physical cues have received less attention from researchers. Yet, temperature and humidity were shown to be detected by *V. destructor* [29]. The size of the brood cell was also highlighted as a criterion that could be important in the host choice behaviour [31]. Because of the difficulty to isolate *V. destructor* from its chemical environment, knowledge about the relevance of physical cues in their foraging and feeding behaviours is still lacking.

Laboratory rearing methods offer a useful tool to try disentangling the role of olfaction from the role of other senses in the honey bee parasite. Several methods to artificially maintain *V. destructor* females in laboratory conditions have been described, some of which do not require the presence of honey bee host [46, 57]. In these artificial conditions, the parasite must move, feed and survive with no or limited help from olfactory molecules naturally occurring within a bee colony. These systems offer a unique opportunity to investigate if *V. destructor* is able to locate a feeding site using senses other than olfaction.

In this study, we aimed to examine whether olfaction is required for the honey bee parasite to find a potential food source in artificial conditions. Firstly, we tested whether *V. destructor* females were able to feed on a synthetic membrane covering a liquid solution based solely on physical cues. More precisely, the importance of parameters such as chemical cues and humidity coming from the food source, the bee or a Parafilm™ membrane was investigated. Secondly, we tested if blocking the parasite major sensory organ impacted their ability to find the artificial food source. Finally, on a shorter timescale and in specifically designed arenas, we recorded and analysed the locomotor behaviour of the mite when facing such artificial food sources. The aim was to elucidate how the liquid feeding solution could be detected by *V. destructor*. The role of physical cues in parasite orientation is explored and discussed in this work.

## Materials and methods

Our studies were conducted according to European ethics laws for scientific research currently in force (Directive 2010/63/EU of the European Parliament and the Council of 22 September 2010 on the protection of animals used for scientific purposes).

### Biological material

*Varroa destructor* adult females were collected in brood frames from eight *A. mellifera* colonies kept on the university campus (INU Champollion, Albi, France) from March to September 2021 and 2022. Briefly, sealed brood frames containing late pupal stages (light to dark pigmentation of the body) were taken to the laboratory. At this stage, the mites are morphologically and physiologically close to dispersal mites and suitable to study survival and locomotion, compared to early reproductive swollen mites. The cells were opened with tweezers and *V. destructor* females were collected. The foundresses were then kept in a Petri dish on lightly to darkly pigmented pupae for 2 h (34 °C, 20% RH) until the start of the experiment. The same sampling method was used for artificial feeding and behavioural bioassays.

### Artificial feeding experiments

#### Feeding chambers

**Figure 1:**
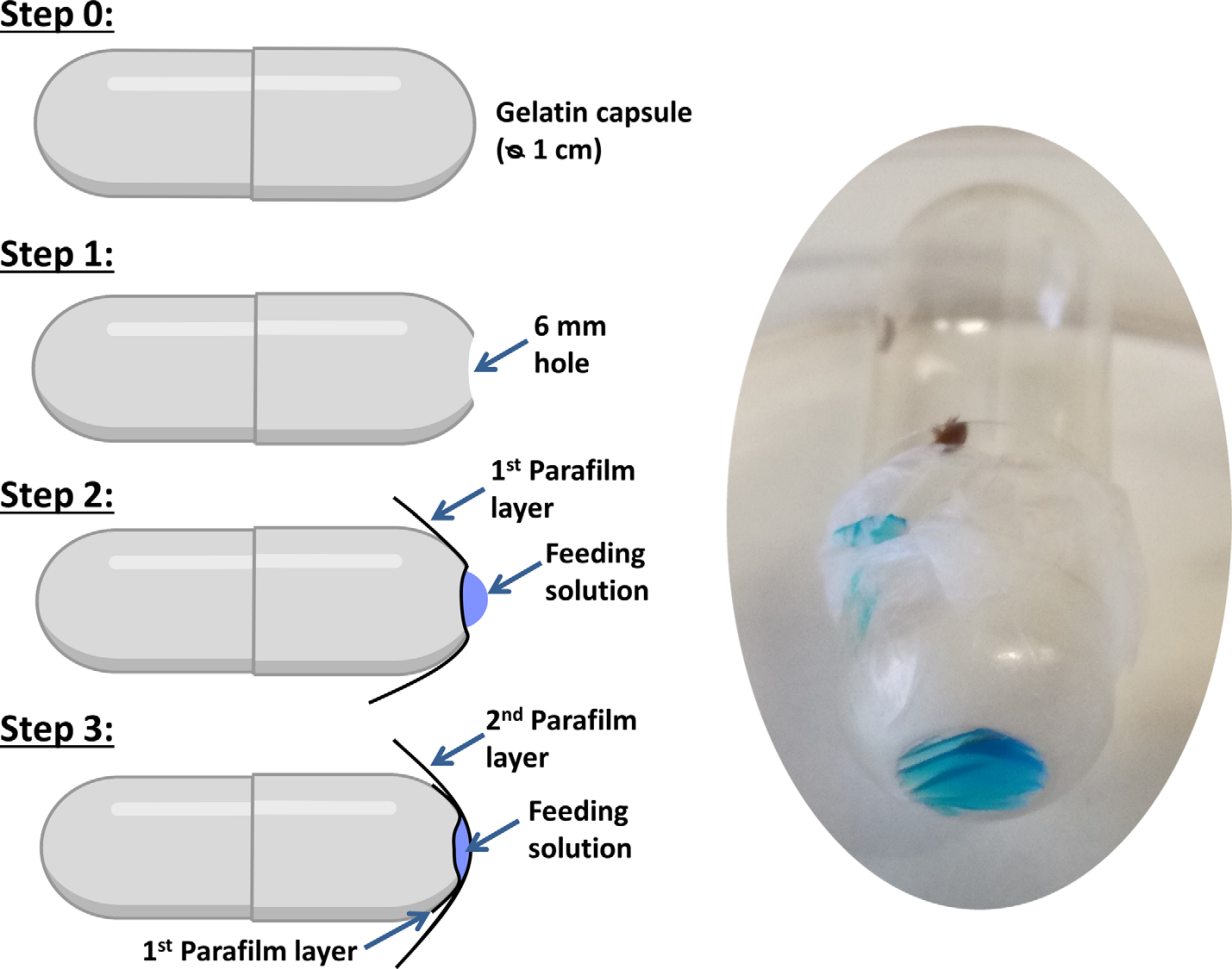
*Experimental set up used in the artificial feeding experiments*. The feeding chambers are made from a gelatine capsule and Parafilm™ membranes. FCF blue dye is included in the nutritive solution to allow for the observation of *V. destructor* feeding status.

To make the artificial feeding chambers, the methodology described in Posada-Florez *et al.* (2020) [46] was followed, with gelatine capsules rather than plastic microtubes (Fig. 1). First, a 6 mm hole was shaped in 1.37 mL gelatine capsules heads (LGA, La Seyne-sur-Mer, France). Two breathing holes were pierced with a needle in the body of the capsules. Sterilisation of the artificial chamber for 20 min under a UV light was then performed. Under a filtering fume hood, a 4 × 4 cm piece of Parafilm™ (Bemis, Co., Inc., Oshkosh, WI, USA) was fully stretched to around 16 μm thick as in Posada-Florez *et al.* (2020) [46] and Ramsey *et al.* (2019) [50]. It was then applied directly onto the 6 mm hole to create a small Parafilm well (Fig. 1, step 2). Twenty microliters of an FCF blue dyed nutritive solution were deposited in this well. A second membrane of Parafilm was used to seal the well and create a bubble containing the nutritive solution (Fig. 1). Similarly to Ramsey *et al.* (2019), a pigmented bee pupa was rubbed on the outer surface of the bubble before introduction of *V. destructor* females in the artificial chamber. In our study, two parasites were introduced in the capsule and the sealed capsule was transferred to an incubator for 48 h (34 °C, 70% RH). After 24 h in the artificial chamber, the feeding status and survival of the parasite were checked. The feeding success was assessed by the detection of blue dye inside *V. destructor* gut either externally or through the dissection of the parasite [44]. The survival status was also noted at 48 h.

#### Parameters tested in the feeding chamber

**Figure S1:**
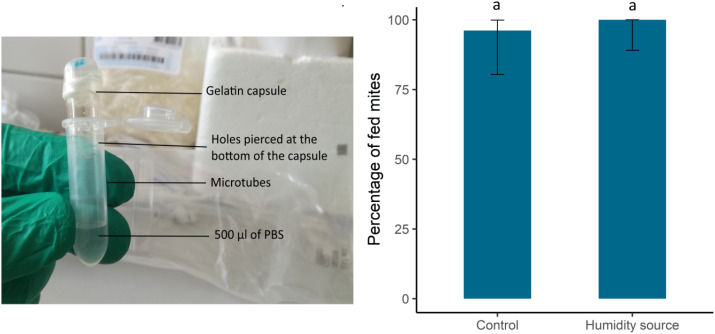
Effect of an alternative source of humidity on the ability of *Varroa destructor* to find the food source.

In this set of experiments, the general methodology to make artificial chambers was followed (Fig. 1), but some parameters were modified to determine their importance in the detection of the feeding site. The control nutritive solution consisted of a mix of cell culture media, as described in Posada-Florez *et al.* (2020) [46] (30% Schneider’s medium, 30% CMRL-1066, 15% blue dyed sterile water, 10% foetal bovine serum, 10% TC-100 insect medium, 4% Insect medium supplement and 1% Hanks salt solution). In a second group, the solution remained the same but the importance of olfactory cues coming directly from the honey bee was addressed by suppressing the rubbing of a bee pupa on the outer surface of the Parafilm membrane (Table 1). As Parafilm can be porous to small volatile molecules coming from the nutritive solution, the complex nutritive medium was replaced by a PBS solution coloured with FCF Blue dye in a third group (5 μL of 1% Blue FCF solution (Vahiné, Avignon, France) in 1 mL of PBS). A fourth group in which the walls of the capsule were entirely covered with Parafilm was also tested. In this condition, the parasite was surrounded by Parafilm and could not rely on putative olfactory cues coming directly from it. The negative control consisted of *V. destructor* females in gelatine capsules containing only a piece of Parafilm, without any food or liquid. Although the Parafilm membrane is waterproof, the impact of an alternative source of humidity was also tested in these artificial conditions (Supplementary Figure S1). The survival and feeding success of the parasite were checked, as previously described.

**Table 1:** Experimental groups from the artificial feeding experiment with parameters tested and sample sizes. The sample sizes are higher for three of the groups because they were used to assess the survival rates of the parasite.

	Feeding solution	Rubbing of bee on Parafilm™	Chamber walls	Number of mites (*N*)
Control feeding	Cell culture medium*	Yes	Gelatine capsule	140
Control feeding w/o rub	Cell culture medium*	No	Gelatine capsule	32
PBS	PBS 1X	No	Gelatine capsule	94
PBS + Parafilm	PBS 1X	No	Gelatine capsule covered with Parafilm	28
Negative control	None	No	Gelatine capsule	82

* = as in Posada-Florez *et al.* (2020); w/o = without.

#### Deprivation of *V. destructor* sensory pit

In this experiment, *V. destructor* sensing was impaired by covering the tips of the forelegs with nail polish, as in previous studies [20, 38]. Black nail polish (Bérangé, Paris, France) was applied on cold anaesthetised female mites using entomological pins. This experiment enables blocking of the sensory pit organ, involved in odour, temperature and humidity detection [10, 38]. To control the effect of nail polish, a second group of females with a nail polish mark on the dorsal scutum was also tested. Two mites from the same treatment group were introduced in each feeding chamber containing a blue dyed PBS bubble. The feeding status of the parasites was noted after 24 h in both groups (*n* = 44 in the test group and *n* = 37 in the control marked group).

### Behavioural observations

To further investigate the ability of *V. destructor* to detect an artificial feeding site, the parasite’s locomotor response to a liquid covering membrane was analysed on a shorter timescale. These behavioural observations were complementary to the 24 h feeding experiment as they can be used to determine whether the finding of the feeding site was the result of a specific foraging behaviour.

#### Design of testing arenas

**Figure 2:**
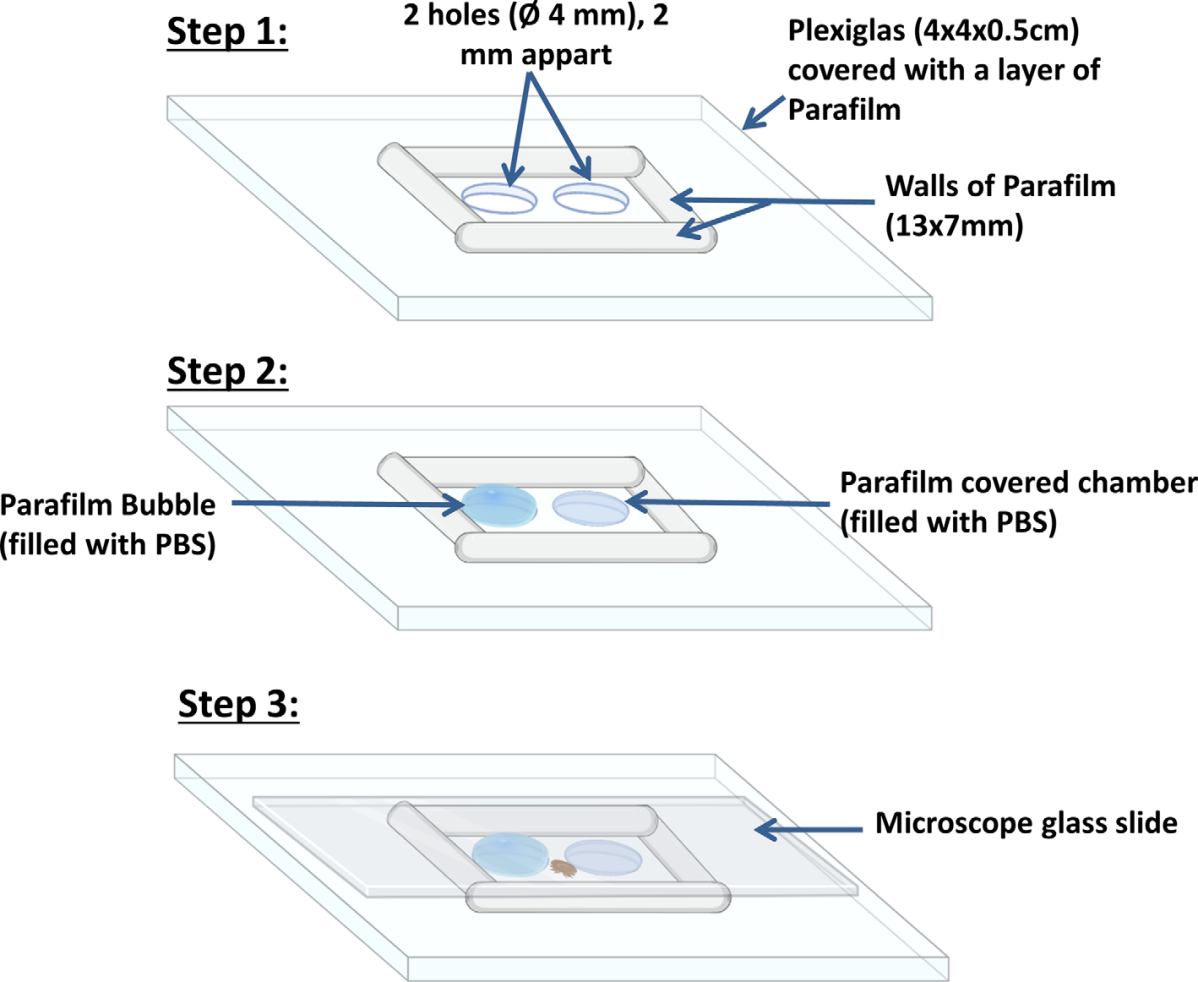
*Steps followed to make the experimental arena used in the behavioural bioassays.* The 13 × 7 × 2 mm arena is covered with Parafilm. A bubble and a discoid membrane covered well are formed. Both contain the same Blue FCF dyed PBS solution. The tested female *V. destructor* is placed in the arena between the second and the third step, right before the start of the recording.

The 13 × 7 mm arenas were made from Plexiglas square pieces (0.5 × 4 × 4 cm). First, two 4 mm wide holes were pierced through the Plexiglas, 2 mm apart from one another (Fig. 2). A first hole was used to generate a bubble of Parafilm filled with blue dyed PBS and the second one to make a membrane-covered chamber filled with the same PBS solution (Fig. 2). The surface of Plexiglas square was also covered with a thin layer of Parafilm to create a homogenous surface with low electrostatic reactions, as they were previously shown to impact *V. destructor* [43]. The Parafilm was always stretched to reach a thickness of around 16 μm [46, 50].

**Figure S2:**
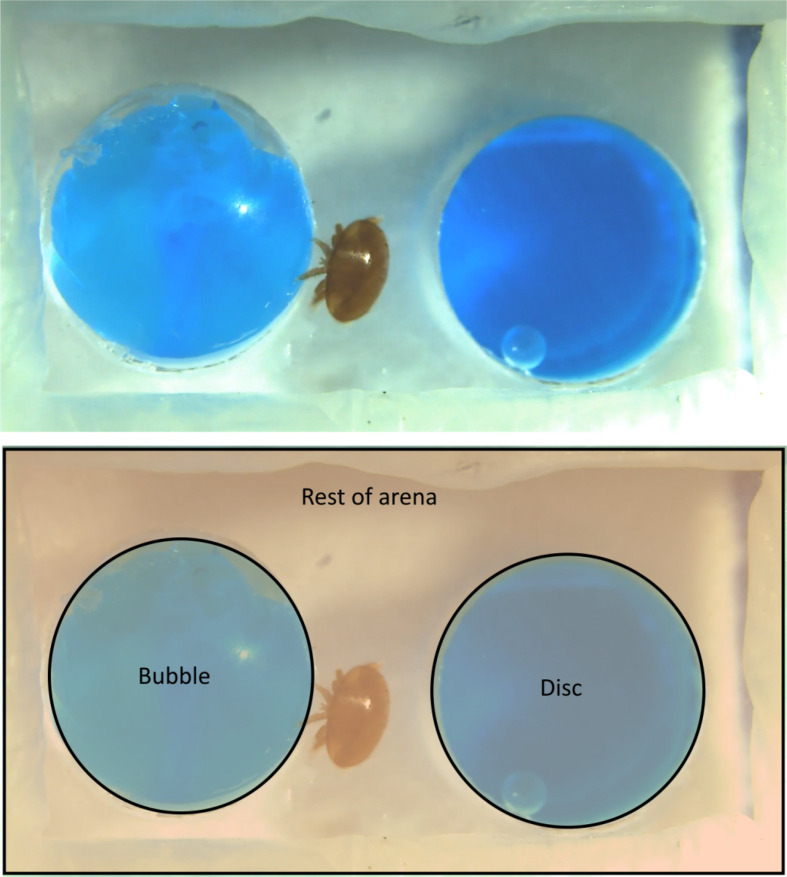
The behavioural bioassay arena from above.

Four Parafilm walls (2 mm high) were then added to define the 13 × 7 mm arena (Fig. 2). A microscope glass slide was finally placed on the top of the walls to seal the arena, while allowing the clear observation of *V. destructor* females from above. Together, these components formed the final set up that was placed under a stereomicroscope to record the parasite’s locomotor behaviour (Fig. 2). The arena was thus composed of three zones. The first two zones, the disc and the bubble were named after the shape of the Parafilm membrane covering the PBS solution. A third larger zone, referred to as rest of the arena, included the floor and the walls (Supplementary Figure S2).

#### Recording and behavioural analyses

The arena was placed under a stereomicroscope (Leica S8APO, Leica Microsystemes, France), which was connected to a camera (Leica MC 190HD) and a screen (Iiyama). A female adult *V. destructor* was inserted in the arena and its movements were recorded for 10 min. The videotracking of the parasite over 10 min was then analysed using EthoVision^®^ XT 17 (Noldus Information Technology BV, Wageningen, Netherlands [40]). The proportion of time spent in each zone (bubble, disc or rest of the arena) along with the number of stops, the duration of stops and the velocity were all measured directly by the software. Based on preliminary observations, the females were considered immobile if they moved less than 0.2 mm between two track points. When the parasite was immobile, short but clear vibrations or pulsations of its body were frequently observed (Movie S1). These pulsating behaviours appeared to be similar to those described by Hall *et al.* 2022 [19]. We estimated their frequency by counting the number of times the behaviour was expressed in a zone and weighting it by the time spent in each zone.

### Statistical analyses

The results were analysed using R.4.0.4 [49] and graphs were generated using the ggplot2 package [68].

Generalised linear models (GLMs) with Binomial distribution were used to analyse the binary data resulting from the artificial feeding experiments. The feeding success and the survival rate at 24 and at 48 h were compared according to the rearing condition of the *V. destructor* females. If significant differences were detected, the groups were further compared using the multcomp package in R. Some conditions resulted in 100% fed female mites, so the parasite feeding rate was analysed using a bias-reduced GLM.

The parameters considered in the videotracking experiments were measured per zone of the arena. Because in the case of random movements, the probability to be in a zone is proportional to the surface of the zone, our measurements of stops and durations by zone had to be weighed by the surface of each zone. The analyses thus focus on these ratios that should not differ if the movements and behaviours of the parasite in the arena are random. When the residuals allowed it – namely in the case of the number of stops and number of pulses – linear models were performed and *post-hoc* analyses were run to further explore the differences. Alternatively, for the proportion of time spent in each zone and the duration of stops, Kruskal–Wallis analyses were used and paired data Wilcoxon tests allowed us to further assess the pairwise differences between zones. The significance threshold was 0.05, except in pairwise analyses for which Bonferroni corrections had to be applied.

## Results

### Effect of the restriction of chemical cues on *V. destructor* feeding success

#### Feeding in control and modified chambers

**Figure 3:**
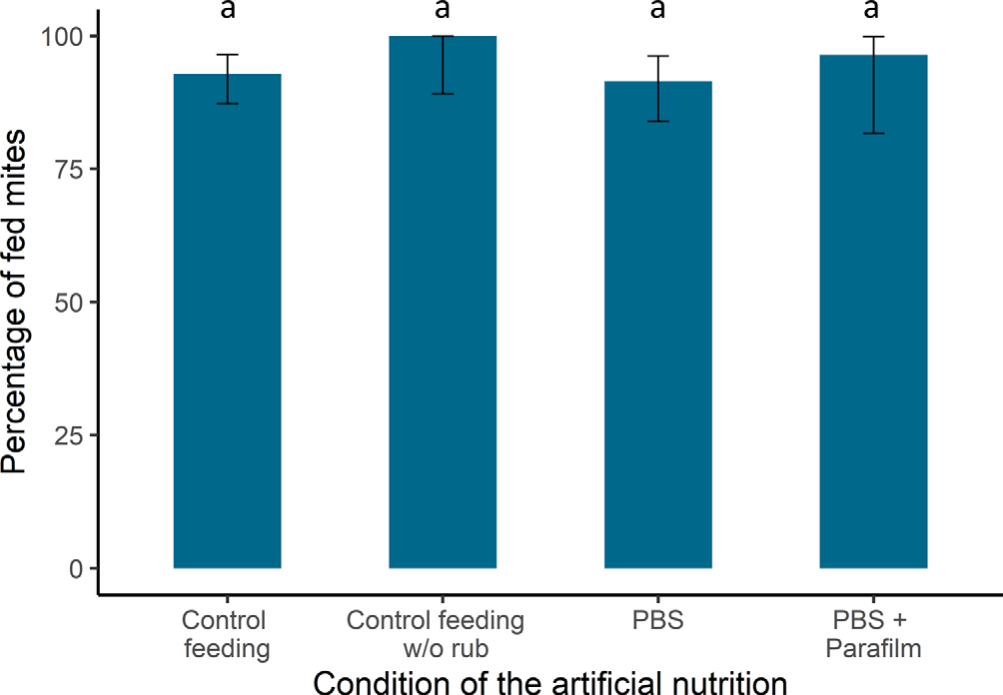
*Feeding success of artificially fed* V. destructor *females*. Percentage and 95% conf. interval of females that had fed 24 h after the start of the experiment, in relation to the experimental set up used (Control feeding (*n* = 140), Control feeding w/o rub (*n* = 32) PBS (*n* = 94) PBS + Parafilm (*n* = 28). Letters indicate the statistical significance between groups.

The feeding success was assessed by the detection of Blue dye inside *V. destructor* gut either externally or through the dissection of the parasite. The feeding success rates remained high regardless of the treatment and no significant differences were found between them (bias-reduced GLM, df = 3, χ^2^ = 4.25, *p* = 0.24). In the control condition, *V. destructor* females were able to detect the feeding site and to ingest the solution through the membrane in more than 90% of the cases (92.9%, *N* = 140, 95% conf. interval: 87.3–96.5; Fig. 3). When no honey bee odour was present in the system, the parasite was still able to find the food source and feed on the artificial medium (100%, *N* = 32, 95% conf. interval: 89.1–100). The food solution or the Parafilm membrane could represent sources of olfactory cues as well. However, in our study, *V. destructor* females were still able to feed when surrounded by Parafilm (96.4%, *N* = 28, 95% conf. interval: 81.7–99.9), when the food source is replaced by a simple saline solution (93.5%, *N* = 94, 95% conf. interval: 86.3–97.6) or when an alternative source of humidity is present (Supplementary Data S1).

#### Survival in control and modified chambers

**Figure 4:**
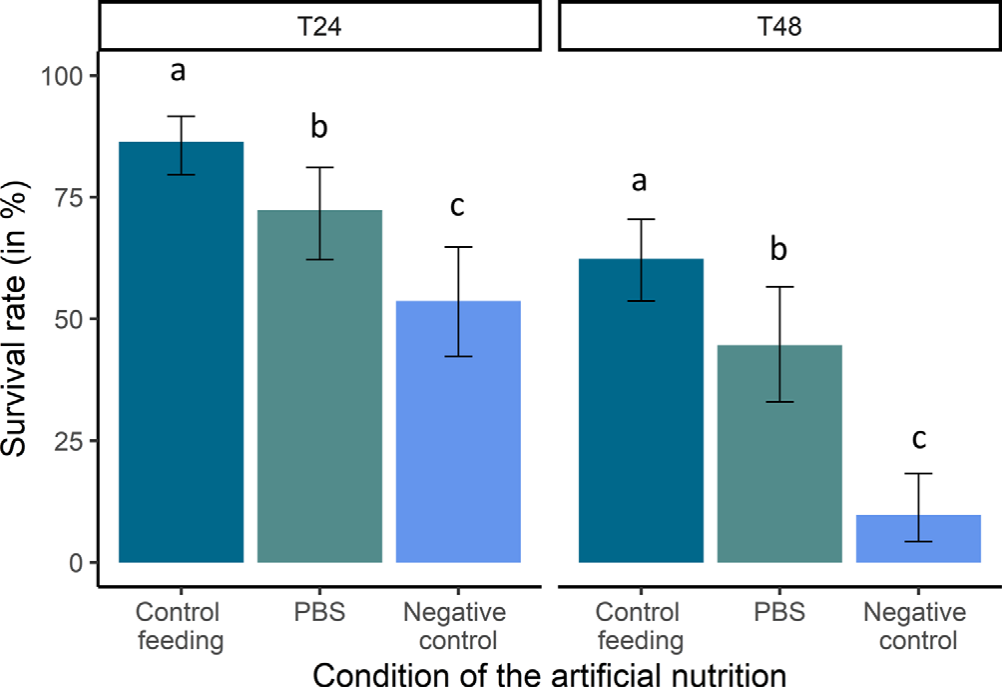
*Survival of* V. destructor *females in artificial conditions*. Survival rates and 95% conf. interval of females (in %) 24 h and 48 h after the start of the experiment, in relation to the experimental set up used (Control feeding *n* = 140; PBS *n* = 94; Negative control *n* = 82). Letters indicate the statistical significance between groups.

Twenty-four hours after the start of the experiment, the survival rate of *V. destructor* females fed on the artificial nutritive solution (positive control) was high (86.4%, 95% conf. interval: 79.6–91.6) but decreased to 62.3% the following day (Fig. 4). Starved females with no access to any food source survived for 24 h in 53.7% of cases (95 conf. interval: 42.3–64.7), but the survival then dropped to 9.8% after 48 h (95% conf. interval 4.3–18.3). Parasites can survive for a limited amount of time by ingesting only a PBS solution. The survival rate of these females was indeed significantly higher than the negative control and lower than the positive control (Fig. 4). This effect was observed at 24 (72.3% 95% conf. interval: 62.2–81.1) and 48 h (44.6% 95% conf. interval: 33.0–56.6). The differences between the three groups were significant for both times considered (T24 h GLM df = 2, χ^2^ = 28.6, *p* < 0.001; T48 h GLM df = 2, χ^2^ = 65.1, *p* < 0.001).

#### Feeding of sense-deprived females *V. destructor*

**Figure 5:**
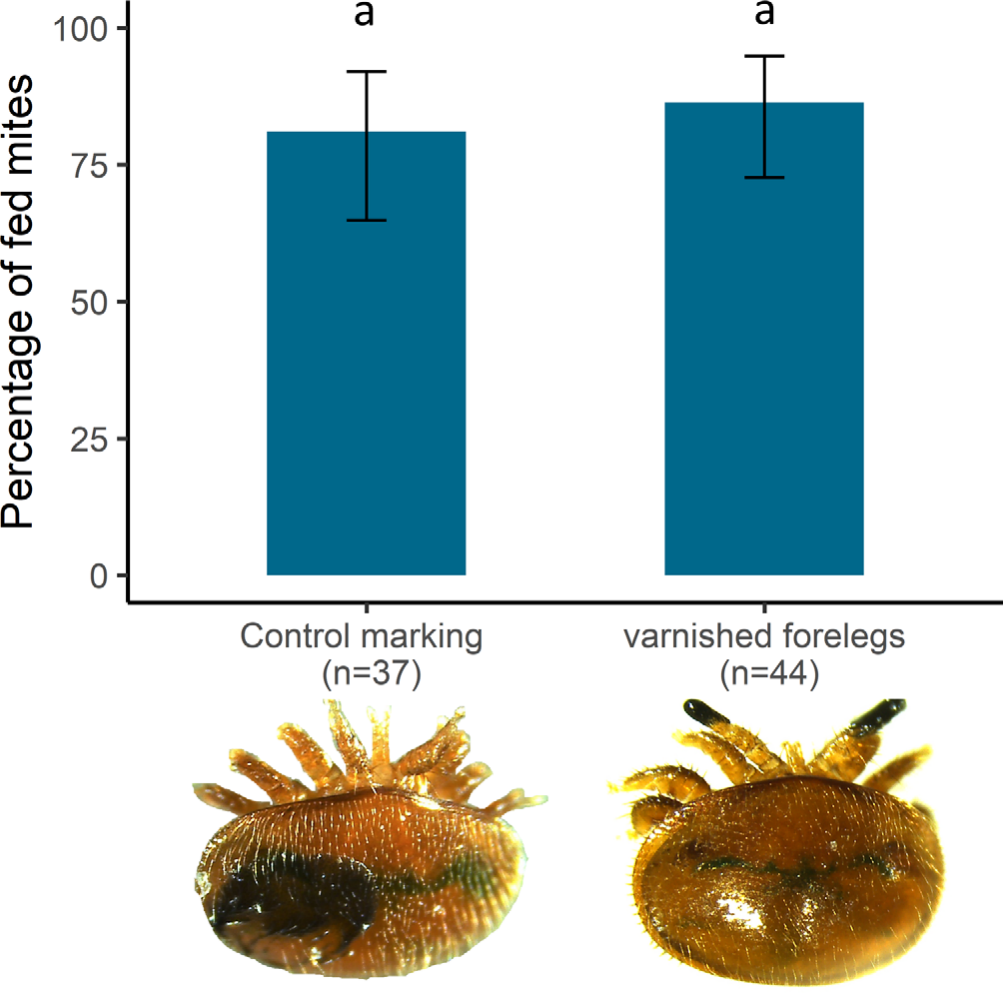
*Effect of sensory pit organ deprivation on the ability of* V. destructor *to find a food source in artificial conditions*. The graph shows the percentage and 95% conf. interval of varnished (*n* = 44) and control (*n* = 37) parasites that fed after 24 h in artificial conditions. Letters indicate the statistical significance between the two groups.

When the females’ forelegs were covered with nail polish, the feeding success rate was still high, with 86.4% (95% conf. interval: 72.6–94.8) of the parasites that fed after 24 h (Fig. 5). This was not significantly different from the control parasites marked with nail polish on their dorsal scutum (81.1%, 95% conf. interval: 64.8–92.0; GLM Df = 1, χ^2^ = 0.42, *p* = 0.52). The mites were still able to detect the feeding site when deprived of the sensory pit.

### Behavioural observations

**Figure 6:**
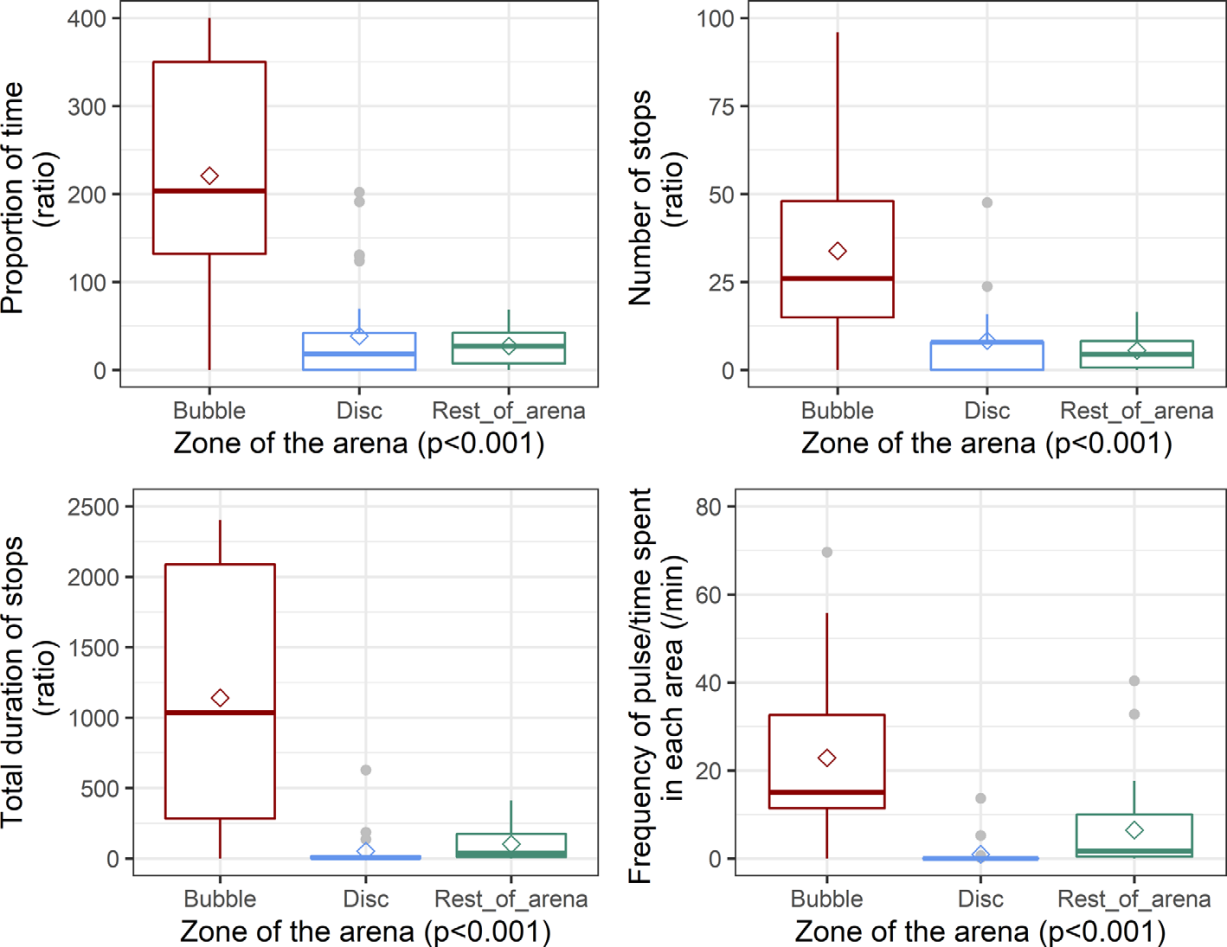
*Behavioural analyses of* V. destructor *females (N = 32) maintained in artificial arenas with a PBS bubble and a PBS discoid membrane*. The first three boxplots show the ratios of the proportion of time spent (in %), the number of stops, the duration of stops (in seconds) weighed by the surface of each zone of the arena (in cm^2^). The frequency of pulse (/min) was also analysed by zone and is displayed on the fourth boxplot. The diamond points show the mean value. The four parameters all differed significantly between the zones considered (Table 2).

The behavioural analyses revealed that the parasites spent a high amount of time on the bubble (mean ± SE: 55.2 ± 6.1% of the 10 min), whereas they mostly ignored the flat Parafilm membrane covering the PBS-filled well (4.9 ± 1.3%, Fig. 6). The amount of time spent on the bubble is actually due to an arresting effect of the hemispheric surface [27], which translates into a reduction or a stop of locomotion when in contact with the bubble surface. Females stopped more often (10.3 ± 1.9) and for longer periods (285.3 ± 40.9 s) when on the bubble compared to the flat membrane (1.9 ± 0.9 and 6.6 ± 3.2 s, Fig. 6). The effect of the spherical surface of the bubble is confirmed by the behaviour of parasites in the rest of the arena. Indeed, the proportion of time spent in the arena (39.9 ± 5.6%) was almost exclusively spent on walls of Parafilm that also happened to have a rounded surface. The number and duration of stops on walls were in between the values of the two other zones (8.1 ± 1.4 and 149.3 ± 30.7 s). To take into account the impact of the zone surface on our results, ratios of parameters weighed by the surface were calculated. The differences are even more striking and the ratios of the amount of time (Kruskal–Wallis df = 2, χ^2^ = 37.9, *p* < 0.001), the frequency of stops (LM *df* = 2, *F* = 12.25, *p* < 0.001) and duration of stops (Kruskal–Wallis *df* = 2, χ^2^ = 42.8, *p* < 0.001) all differed significantly between zones (Fig. 6). Based on *post hoc* analyses, the locomotor behaviour of *V. destructor* in the bubble zone was significantly different from its behaviour in the two other zones (Table 2).

**Table 2:** *Post hoc* analyses of the behaviours of *V. destructor* in experimental arenas (*N* = 32).

	Bubble *vs* rest of arena	Bubble *vs* Disc	Disc *vs* rest of arena
Time spent in zone *	*V* = 506 ***p* < 0.001**	*V* = 462 ***p* < 0.001**	*V* = 236 ***p* = 0.9**
Frequency of stops^a^	***p* < 0.001**	***p* < 0.01**	***p* = 0.5**
Duration of stops *	*V* = 443 ***p* < 0.001**	*V* = 179 ***p* < 0.001**	*V* = 34 ***p* < 0.05**
Frequency of pulse^a^	***p* < 0.001**	***p* < 0.001**	***p* = 0.2**

* = paired samples Wilcoxon test, all *p*-values were adjusted following the Bonferroni correction; a = Tukey HSD, all *p*-values were adjusted.

When the females *V. destructor* are immobile on a surface, they can often be seen pulsating as described by Hall *et al.* (2021; Movie S1). The frequency of these pulses was higher in the bubble zone (22.9 ± 3.1 pulses/min) when compared to the other two zones (0.9 ± 0.7 pulses/min on the membrane and 6.5 ± 1.7 pulses/min in the rest of the arena). In the rest of the arena, most of the pulses (78%) occurred on the Parafilm walls. The pulses occurred significantly more often on the bubble than expected if the probability of pulsing would be randomly expressed, in which case it would depend only on the time spent in the zone (Table 2, Fig. 6).

## Discussion

It is well known that in a host-parasite relationship, the parasite will seek cues from its host to find and join it [8]. In its natural environment, the behaviour of the eyeless parasite *V. destructor* is highly influenced by semiochemicals emitted by honey bees [35]. Previous studies exploring the effect of olfaction on parasite feeding behaviour have highlighted their key role in the parasite lifecycle [38, 42]. Similarly, in artificial feeding experiments without honey bees, host kairomones were thought to be important for parasite orientation. Usually, the rubbing of a bee on the feeding site was thus recommended to leave useful olfactory cues and guide *V. destructor* towards the food source [50].

Our data suggest that in artificial conditions, olfactory cues from bees may not be essential for the parasite to find a food source. Artificial feeding of *V. destructor* foundresses can be achieved with about 90% success in the laboratory, without a need for chemical cues. This puts into perspective the crucial importance of honey bee olfactory cues, at least in laboratory conditions.

In the absence of honey bee kairomones, *V. destructor* could still rely on other sources of odours to detect the food source. Olfactory cues emitted by the feeding medium and/or the Parafilm membrane could for instance be used for the parasite orientation. In this work, we showed that a feeding medium containing only PBS can lead to a feeding success in 96% of the cases. Similarly, when the parasite is surrounded by Parafilm, its ability to find the specific feeding site remains high (around 93%). The significantly higher survival rate of females kept on a PBS medium when compared to unfed females is also interesting as it could be a convenient negative control in future laboratory rearing experiments. More strikingly, when deprived from its main olfactory organ (*i.e.*, the sensory pit on the forelegs) [10], the parasite is still able to find the food source and feed through the membrane. By preventing *V. destructor* from relying on environmental odours to forage, we provide evidence of artificial feeding with no or a limited role of olfaction. If the parasite is able to locate a food source without the help of olfactory cues, the process underlying this capacity remains an enigma.

In some liquid sucking parasitic insects such as aphids, it is known that foraging depends on a trial-and-error (probing) strategy [47, 59]. In *V. destructor*, the searching of the feeding site in natural conditions seems much more oriented and the female does not appear to bite randomly. When it feeds on pupae, a single feeding site located on the ventral side of the abdomen is made [11, 26]. On adults, several feeding sites are observed but they are also concentrated on the ventro-lateral side of the abdomen [50]. In artificial conditions, although a probing behaviour cannot be ruled out, the parasite appears more likely to rely on non-olfactory tactile or physical cues. Thanks to thermo and hygroreceptors located on the forelegs, it is known that *V. destructor* is able to detect humidity or heat [10, 29]. The existence of other receptors and the exact role of the foreleg sensilla in the process, however, remain unclear. In our experiments, temperature was kept constant in the gelatine capsule and the parasite’s orientation did not appear to depend on it. Regarding humidity, Parafilm is considered a waterproof membrane and an alternative source of humidity does not affect the mite’s feeding success. Furthermore, our behavioural bioassay where parasites discriminated between two similar membranes covering the same PBS solution shows that humidity is not an essential factor in this context. In contrast, our results provide support for the hypothesis that the parasite uses physical cues to locate food sources. During our 10-minute behavioural tests, the shape of surfaces seems to be recognised by *V. destructor* females as they are able to detect a 3D shaped membrane and explore it more extensively than the alternative 2D membrane. The time spent, number and duration of stops are all higher on the Parafilm bubble than in the rest of the arena, especially if we consider the reduced surface of the bubble. The Parafilm bubble thus seems to have an arrestant effect on the parasite. In this case, the parasite would first walk randomly in the arena and as the unexplored space is reduced, would finally find the food source. The arrestant effect of the 3D rounded aspect would lead *V. destructor* females to stay on this “larval like” shape. In arthropods, the tactile sense has rarely been studied in comparison with vision, olfaction or hearing, although it seems to be highly developed in several Arachnid species [3]. It relies on mechanoreceptors that are widely abundant on their hairy integument [3, 54, 64]. The tactile sense was shown to be essential for reproduction, orientation or hygiene in mites and spiders [4, 53–55]. In the two-spotted spider mite for instance, shape of quiescent deutonymphs seems to trigger the arrestment behaviour of reproductive males [53]. In our study, female *V. destructor* expressed the same type of arrestment when in contact with a rounded surface covering a liquid. The shape of the feeding site thus seems crucial. If it is not adequate or too flat, the female will spend less time exploring, which would result in an inability to feed. Since females explored the bubble more extensively than the 3D rounded walls of the arena, they could also have detected that the Parafilm membrane is thinner on the bubble and that it covers a liquid solution, which would make it more suitable for feeding.

In natural conditions, the ability to assess shapes and surfaces in dark and odour-saturated sealed brood could be beneficial for the mite to find its way around the cell and feed on the bee pupa. The behavioural mechanisms behind this capacity, however, remain unknown. Hall *et al.* (2022) recently described an unknown pulsating behaviour expressed by *V. destructor* females when they are exploring their environment [19]. We also observed this pulsating behaviour (Movie S1) and quantified it. We highlighted a higher frequency on 3D membrane compared to the rest of the arena, even when the total amount of time spent in each zone was taken into account. This means that once on the membrane, *V. destructor* spent less time moving and more time pulsating. The fact that pulsations are more expressed on liquid covering curved membranes could indicate that it is a crucial behaviour making it possible to gather physical cues from the environment, at least in the absence of olfactory cues. Further experiments are needed to examine whether *V. destructor* expresses this vibrational behaviour to assess the suitability of the feeding site before engaging its feeding behaviour [30, 48].

In any case, *V. destructor* would not be an exception as many other arthropods are known to vibrate. Biotremology is indeed a whole field in biology that highlights the importance of multimodal signals in animal behaviour, with a particular attention to the neglected aspect of vibrational communication and orientation [21]. Again, many arachnid species are well known to use vibrations in their foraging [5, 15, 30, 61]. Scorpions even possess an anatomical structure, namely the basitarsal compound slit sensilla that was shown to act as a vibrational receptor [67]. To our knowledge however, no similar structure has been described in *V. destructor* but it would be interesting to explore it further.

Altogether, our results obtained in an artificial context put into perspective the relative importance of olfactory and tactile senses in *V. destructor*. Whether the parasite’s ability to detect different shapes is always expressed or only in critical conditions remains to be determined. The artificial feeding method offers a controlled environment which allows for the exploration of hypotheses that are difficult to test in natural conditions. Unexplored aspects of parasite biology, like its nutritional requirements and foraging behaviour, could indeed be investigated through laboratory experiments. The consistent ingestion of artificial food from 3D Parafilm dummies is also a first step in the development of a long-term rearing method for *V. destructor*, using synthetic or semi-natural feeding solutions. However, if nutrition can be triggered with only a few or no olfactory cues, this is unlikely to be the case for reproduction, and recreating the whole parasite cycle in laboratory conditions remains the next big challenge.

## Supplementary materials

The Supplementary materials of this article are available at https://www.parasite-journal.org/10.1051/parasite/2023049/olm.

*Video S1-a*: Observation of *Varroa destructor* females’ pulsating behaviour under a stereomicroscope – dorsal view (×10). *Video S1-b*: Observation of *Varroa destructor* females’ pulsating behaviour under a stereomicroscope – lateral view (×40). *Video S1-ci*: Observation of *Varroa destructor* females’ pulsating behaviour under a stereomicroscope – ventral view (×20)
